# Fracture Risk in Trans Women and Trans Men Using Long‐Term Gender‐Affirming Hormonal Treatment: A Nationwide Cohort Study

**DOI:** 10.1002/jbmr.3862

**Published:** 2019-10-07

**Authors:** Chantal M Wiepjes, Christel JM de Blok, Annemieke S Staphorsius, Nienke M Nota, Mariska C Vlot, Renate T de Jongh, Martin den Heijer

**Affiliations:** ^1^ Department of Endocrinology Amsterdam UMC, VU University Medical Center Amsterdam the Netherlands; ^2^ Center of Expertise on Gender Dysphoria, Amsterdam UMC, VU University Medical Center Amsterdam the Netherlands

**Keywords:** BONE, FRACTURES, GENDER‐AFFIRMING HORMONAL TREATMENT, OSTEOPOROSIS, TRANSGENDER

## Abstract

Concerns about bone health in transgender people using gender‐affirming hormonal treatment (HT) exist, but the fracture risk is not known. In this nationwide cohort study, we aimed to compare the fracture incidence in transgender people using long‐term HT with an age‐matched reference population. All adult transgender people who started HT before 2016 at our gender‐identity clinic were included and were linked to a random population‐based sample of 5 age‐matched reference men and 5 age‐matched reference women per person. Fracture incidence was determined using diagnoses from visits to hospital emergency rooms nationwide between 2013 and 2015. A total of 1089 trans women aged <50 years (mean 38 ± 9 years) and 934 trans women aged ≥50 years (mean 60 ± 8 years) using HT for median 8 (interquartile range [IQR] 3–16) and 19 (IQR 11–29) years, respectively, were included. A total of 2.4% of the trans women aged <50 years had a fracture, whereas 3.0% of the age‐matched reference men (odds ratio [OR] = 0.78, 95% confidence interval [CI] 0.51–1.19) and 1.6% of the age‐matched reference women (OR = 1.49, 95% CI 0.96–2.32) experienced a fracture. In trans women aged ≥50 years, 4.4% experienced a fracture compared with 2.4% of the age‐matched reference men (OR = 1.90, 95% CI 1.32–2.74) and 4.2% of the age‐matched reference women (OR = 1.05, 95% CI 0.75–1.49). A total of 1036 trans men (40 ± 14 years) using HT for median 9 (IQR 2–22) years were included. Fractures occurred in 1.7% of the trans men, 3.0% of the age‐matched reference men (OR = 0.57, 95% CI 0.35–0.94), and 2.2% of the age‐matched reference women (OR = 0.79, 95% CI 0.48–1.30). In conclusion, fracture risk was higher in older trans women compared with age‐matched reference men. In young trans women, fracture risk tended to be increased compared with age‐matched reference women. Fracture risk was not increased in young trans men. © 2019 The Authors. *Journal of Bone and Mineral Research* published by American Society for Bone and Mineral Research.

## Introduction

Transgender people can receive gender‐affirming hormonal treatment (HT) to change the physical characteristics belonging to their experienced gender. Trans women (birth‐assigned males, female identity) receive estrogens to induce feminization, resulting, for example, in breast growth^1^ and changes in body composition.^2^ Trans men (birth‐assigned females, male identity) are treated with testosterone, which, among others, stimulates lowering of the voice^3^ and growth of body hair.^4^

Sex steroids also play important roles in the acquisition and homeostasis of bone. In cis (non‐transgender) men, testosterone stimulates periosteal apposition, leading to a wider cortical bone size than in women,^5^, ^6^ whereas in cis women, estrogen stimulates endosteal bone formation.^7^ In women, loss of estrogen at menopause leads to an increase in osteoclast activity and a decrease in bone mineral density (BMD).^8^, ^9^ However, in women with hyperandrogenism, higher BMD was found,^10^ whereas in women with complete androgen insensitivity syndrome, lower BMD was found.^11^ These findings indicate that, besides estrogen, testosterone also regulates BMD in women. In men with aromatase deficiency, lower bone mass was observed.^12^, ^13^, ^14^ In addition, a decrease in BMD was found in men treated with aromatase deficiency with normal testosterone concentrations,^15^ indicating that estrogen is also an important determinant of bone health in men.

Besides changes in physical characteristics in transgender people, HT also influences BMD. Earlier studies found either a maintenance^16^, ^17^, ^18^, ^19^, ^20^ or increase^16^, ^17^, ^18^, ^21^, ^22^, ^23^, ^24^, ^25^, ^26^ in BMD in both adult trans women and trans men after short‐term HT. Long‐term effects of HT have been investigated in a few small‐sample cross‐sectional studies in which trans women were compared with control men, and trans men with control women. Contradictory results were obtained from these studies: higher,^27^, ^28^ similar,^28^, ^29^ and lower^30^ BMD than controls were found. One follow‐up study found no change in BMD in trans women and trans men during the first 10 years of HT.^31^ However, before the start of HT, trans women were found to have relatively low BMD, possibly because of co‐existing vitamin D deficiency and a different lifestyle, leading to decreased muscle mass and therefore decreased mechanical loading on bone.^32^

In clinical practice, there are concerns about bone health in transgender people, particularly regarding the low initial BMD in trans women and the lack of fracture data. A few studies described no increased fracture risk before the start of HT in trans women^24^, ^32^ and trans men.^20^ In short‐term follow‐up studies, no fractures were observed in trans women,^24^ trans men,^20^ or their controls. In studies after long‐term HT, no increased fracture risk was found in both trans women^30^ and trans men.^33^ However, all these studies had a small sample size (*n* < 50) or were using questionnaires to define fractures.

Therefore, the aim of this study is to investigate the fracture incidence in a large cohort of adult trans women and trans men after long‐term HT, based on diagnoses from visits to the hospital emergency rooms nationwide, and to compare this incidence with an age‐matched male and female reference population. In addition, we aimed to study whether the types of fractures differed between the transgender population and their age‐matched reference groups and whether BMD or other characteristics were different in the transgender population with fractures compared with those without fractures.

## Materials and Methods

### Study design and population

This study is part of the Amsterdam Cohort of Gender Dysphoria study,^34^ including all 6793 people who once visited the gender‐identity clinic of the Amsterdam University Medical Center, the Netherlands, between 1972 and 2016. Study design and population have been described previously.^34^ In short, the medical files of these people were reviewed and clinical data were retrieved. For the current study, only people who started with HT and were not deceased at time of data collection were included. The cohort was linked to a random sample of 5 age‐matched males and 5 age‐matched females per transgender person, provided by the Statistics Netherlands (Central Bureau of Statistics) based on the National Civil Record Registry. The study was approved by the Medical Ethics Committee of the Amsterdam University Medical Centers, location VUMC, and necessity for informed consent was waived because of the retrospective design and the absence of interventions.

### Treatment

After the diagnostic process, people could start with HT when the diagnosis gender dysphoria was confirmed.^35^ In trans women, HT consists of anti‐androgens, which were usually continued until orchiectomy, in combination with estrogens. The most commonly prescribed estrogens were 17‐beta estradiol implants (20–40 mg per 3 months), patches (50–150 μg/24 hours twice a week), or oral valerate (2–4 mg daily), and estradiol gel (0.75–1.5 mg daily). In the past, ethinyl estradiol (50–150 μg daily) and conjugated estrogens (0.625–2.5 mg daily) were mostly used. Cyproterone acetate (50–100 mg daily) was most often prescribed as anti‐androgen, which was usually ceased after orchiectomy. In trans men, HT consisted of testosterone only, which includes testosterone gel (25–50 mg daily), intramuscular or oral testosterone undecanoate (1000 mg per 12–14 weeks, or 40–240 mg daily, respectively), or intramuscular testosterone esters (250 mg every 2–3 weeks). After at least 1 year of HT and after the age of 18 years, surgery could be performed, including vaginoplasty with orchiectomy in trans women and hysterectomy with oophorectomy in trans men.

### Fracture data

The total population of transgender people and their 10 age‐matched references were linked to a database from Statistics Netherlands, which stores all diagnoses made by medical doctors from visits to the hospital emergency rooms nationwide based on Diagnosis‐Treatment‐Combination trajectories (DTCs) for specialized medical care. These DTCs are used to calculate the insurance claim, which is sent to the individual's medical insurance company. Health insurance is obligatory for every inhabitant of the Netherlands. The diagnoses were available for the years 2013, 2014, and 2015.

### Dual‐energy X‐ray absorptiometry (DXA)

BMD was regularly measured during patient care at baseline and thereafter every 5 years, independently of the occurrence of fractures. A DXA Hologic Delphi was used, which was updated in July 2004 and replaced in February 2011 by a Hologic Discovery A (Hologic Inc., Bedford, MA, USA). For both machines, the coefficient of variation (CV) was <1%. Phantom calibration allowed for comparison of the absolute BMD values. Absolute BMD values (g/cm^2^) of the lumbar spine (LS) were obtained and *T*‐scores were calculated, based on the birth‐assigned sex reference values of the National Health and Nutrition Examination Survey (NHANES). BMD measurements were available for 77% of the trans women and 84% of the trans men.

### Laboratory measurements

Blood samples were frequently obtained during patient care. When improved quality assays were available, these were implemented and conversion formulas for comparison of the concentrations were generated. Until January 2010, estradiol was measured using a radioimmunoassay (Diasorin, Saluggia, Italy) with an interassay CV of 10% and a lower limit of quantitation (LOQ) of 18 pmol/L. Between January 2010 and July 2014, a competitive immunoassay (Delfia, Wallac, Turku, Finland) was used (interassay CV 10%, LOQ 20 pmol/L). For conversion, the formula Delfia = 1.267*Diasorin–28.87 was used. Since July 2014, LC–MS/MS (VUMC, Amsterdam, the Netherlands) was used (interassay CV 7%, LOQ 20 pmol/L) and the formula LC–MS/MS = 1.60*Delfia–29 was used for conversion. Testosterone was measured using a radioimmunoassay (RIA; Coat‐A‐Count, Siemens, Malvern, PA, USA) until January 2013 (interassay CV 7–20%, LOQ 1 nmol/L), hereafter a competitive immunoassay (Architect, Abbott, Abbott Park, IL, USA) was used (interassay CV 6–10%, LOQ 0.1 nmol/L). Two formulas were used for conversion: Architect = 1.1*RIA+0.2 for testosterone concentrations <8 nmol/L; Architect = 1.34*RIA–1.65 for testosterone concentrations >8 nmol/L. Luteinizing hormone (LH) was measured using an immunometric assay (Delfia) until June 2011 (interassay CV <7%, LOQ 0.5 U/L). After June 2011, an immunometric assay (Architect, Abbott) was used (interassay CV <6%, LOQ 2 U/L), using the formula Architect = 0.91*Delfia–0.01 for conversion. Mean estradiol, testosterone, and LH concentrations per person were calculated by averaging the results from the measurements performed during HT. Laboratory measurements were available for 66% of the trans women and 72% of the trans men.

### Statistical analysis

Characteristics of the transgender population are presented as mean with standard deviation (SD), median with interquartile range (IQR), or percentages. First, the fracture incidence was calculated in trans women, trans men, and the age‐matched reference men and women. Thereafter, logistic regression analyses were performed to calculate odds ratios (OR) with 95% confidence intervals (CI), as an approximation of the relative risk for fractures. As age affects the risk of fractures, the trans women group was divided into two groups (<50 years and ≥ 50 years), and the analyses were repeated for both age groups separately. Fractures were divided into two groups: hip, spine, forearm, and humerus fractures (which are all an approximation of osteoporotic fractures), and other fractures.^36^ Chi‐square tests were performed to investigate whether the type of fractures differed between the groups. To investigate whether age in 2015, age at start of HT, body mass index (BMI), smoking habits, *T*‐score of the LS, estradiol concentrations, testosterone concentrations, and LH concentrations were associated with fracture risk, multivariable logistic regression analyses were performed in the transgender population. To protect the anonymity of the population, data are only shown if more than 10 individuals were present in each group. Subgroup analyses were therefore not performed if less than 20 individuals were present in one group. Analyses were performed using STATA Statistical Software (StataCorp, College Station, TX, USA), version 14.2.

## Results

### Study population

Of the 6793 people who are included in the total cohort, 2726 people were excluded from this study because they did not start with any treatment (yet), 442 started with treatment during adolescence, 15 received alternating testosterone and estradiol treatment, 319 could not be linked to the Statistics Netherlands database (eg, because they were not registered in the Netherlands), and 232 people were deceased. This led to a total study population of 3059 people, consisting of 2023 trans women and 1036 trans men. The characteristics are shown in Table 1.

**Table 1:** Characteristics of the Study Population

	Trans women aged < 50 years	Trans women aged ≥ 50 years	Trans men
No. of people	1089	934	1,036
Age (years) in 2015	38 (9)	60 (8)	40 (14)
Age (years) at start HT	26 (22–33)	40 (31–48)	25 (21–33)
Duration HT (years)	8 (3–16)	19 (11–29)	9 (2–22)
BMI (kg/m^2^) (*n* = 2756)	23.9 (4.2)	25.7 (4.6)	25.8 (4.9)
Smoking, % yes (*n* = 2614)	44.7	49.0	47.8
Gonadectomy, % yes	57.8	80.9	69.8
*T*‐score lumbar spine	−1.13 (1.23)	−0.91 (1.33)	−0.18 (1.19)
*T*‐score total hip	−0.77 (0.85)	−0.67 (0.88)	+0.04 (0.98)
*T*‐score femoral neck	−0.99 (0.94)	−1.19 (0.88)	−0.30 (1.05)
Laboratory			
Estradiol (pmol/L)	211 (132–308)	241 (138–391)	147 (102–205)
Testosterone (nmol/L)	1.2 (0.7–1.4)	1.3 (1.0–1.3)	25.0 (17.1–36.5)
LH (IU/L)	2.2 (0.2–9.7)	3.2 (0.3–8.4)	3.6 (0.9–11.5)

HT = hormonal treatment; BMI = body mass index; LH = luteinizing hormone. Characteristics are shown as mean with standard deviation, median with interquartile range, or percentage. Associations are shown as odds ratios (OR) with 95% confidence intervals (CI).

Laboratory measurements were available for 66% of the trans women and 72% of the trans men.

### Trans women

Fractures occurred in 3.3% of the trans women (*n* = 67) in the 3‐year time period, whereas 2.7% of the age‐matched reference men (*n* = 275) and 2.8% of the age‐matched reference women (*n* = 283) had a fracture. The overall fracture incidence was not increased in trans women compared with age‐matched reference men (odds ratio [OR] = 1.23, 95% confidence interval [CI] 0.93–1.61) nor with age‐matched reference women (OR = 1.19, 95% CI 0.91–1.56). A total of 41.8% of all fractures in trans women was a hip, spine, forearm, or humerus fracture, compared with 26.6% in age‐matched reference men (*p* = 0.014) and 36.0% in age‐matched reference women (*p* = 0.381).

**Figure 1:**
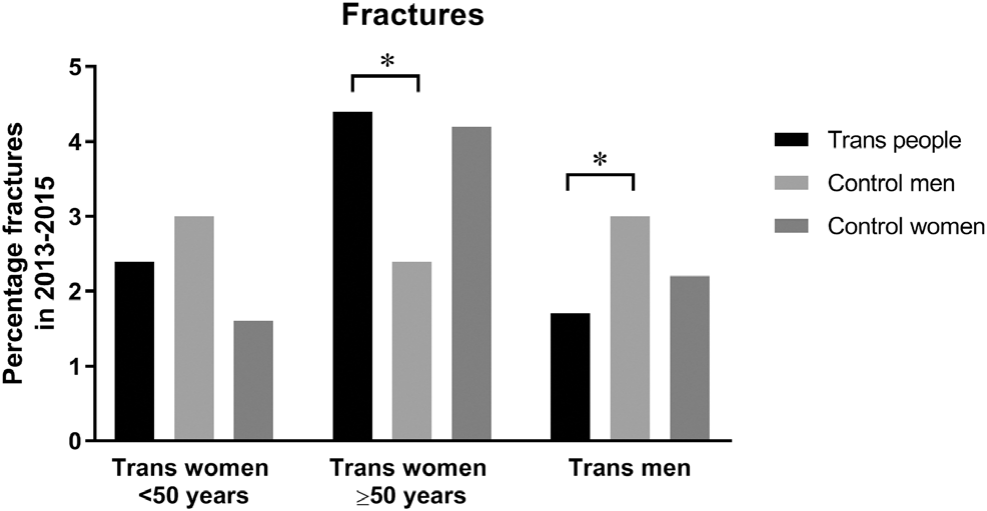
Fracture incidence in the years 2013, 2014, and 2015 in the transgender population and age‐matched reference (control) groups. Data are shown as percentages separately for trans women aged <50 years (black bars), trans women aged ≥50 years (black bars), trans men (black bars), and their age‐matched reference (control) men (light gray bars) and women (dark gray bars). All types of fractures observed at the emergency room during this time frame are shown. Trans women aged ≥50 years have an increased fracture risk compared with age‐matched reference men. Trans men have a decreased fracture risk compared with age‐matched reference men. *p < 0.05. Because of the low number of fractures in trans men, no stratification for age could be performed.

**Figure 2:**
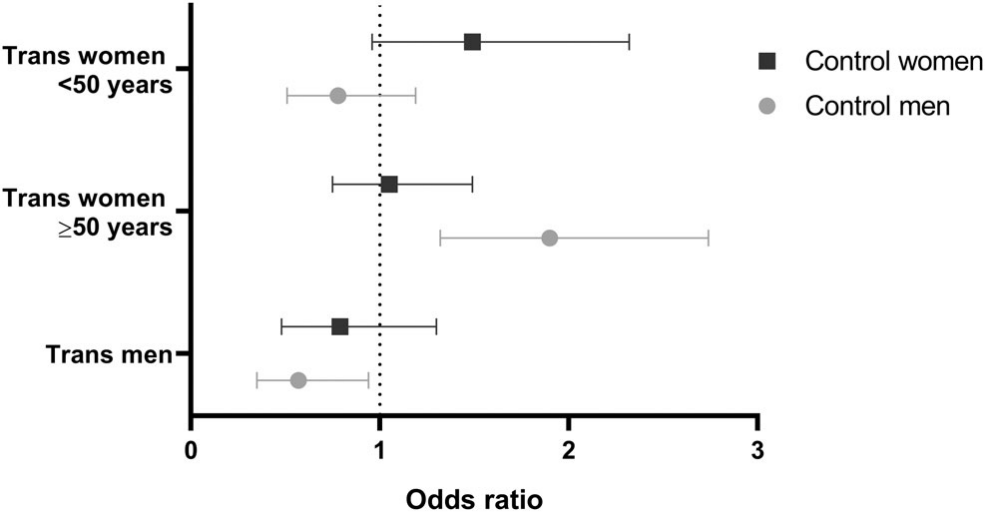
Odds ratios with corresponding 95% confidence intervals for the fracture risk of trans women and trans men compared with age‐matched reference (control) men and women. Odds ratios with corresponding 95% confidence intervals are shown for trans women (aged <50 years or ≥ 50 years) and trans men compared with age‐matched reference (control) women (black lines) and age‐matched control men (gray lines) for all types of fractures observed at the emergency room in 2013, 2014, and 2015. Trans women aged ≥50 years have an increased fracture risk compared with age‐matched reference men. Trans men have a decreased fracture risk compared with age‐matched reference men.

After age stratification, trans women aged ≥50 years (*n* = 934) had an increased fracture risk (4.4%, *n* = 41) compared with age‐matched reference men aged ≥50 years (2.4%, *n* = 110, OR = 1.90, 95% CI 1.32–2.74), but a similar fracture risk compared with age‐matched reference women aged ≥50 years (4.2%, *n* = 195, OR = 1.05, 95% CI 0.75–1.49) (Figs. 1 and 2). Trans women aged <50 years (*n* = 1089) did not have an increased fracture risk (2.4%, *n* = 26) compared with age‐matched reference men aged <50 years (3.0%, *n* = 165, OR = 0.78, 95% CI 0.51–1.19) but tended to have a higher fracture risk compared with age‐matched reference women aged <50 years (1.6%, *n* = 88, OR = 1.49, 95% CI 0.96–2.32) (Figs. 1 and 2).

In Table 2, the differences in characteristics between trans women with and without fractures are shown. In the multivariable analysis, age in 2015 (per year: OR = 1.05, 95% CI 1.02–1.08) and *T*‐score of the lumbar spine (per 1.0 point: OR = 0.75, 95% CI 0.59–0.96) were associated with fracture risk. Smoking tended to be associated with a higher fracture risk (yes versus no: OR = 1.68, 95% CI 0.93–3.04), whereas mean BMI (per point: OR = 1.04, 95% CI 0.97–1.11) and age at start of HT (per year: OR = 0.98, 95% CI 0.95–1.01) were not associated with an increased fracture risk. Laboratory measurements were not included in this multivariable analysis because of the smaller number of individuals with laboratory measurements available. However, in univariable analyses, no associations were found between estradiol (per 10 pmol/L: OR = 0.99, 95% CI 0.97–1.02), testosterone (per 1 nmol/L: OR = 1.03, 95% CI 0.96–1.10), and LH (per 1 IU/L: OR = 1.00, 95% CI 0.97–1.04) concentrations and fracture risk.

**Table 2:** Differences in Characteristics in the Transgender Population With and Without Fractures

	Trans women			Trans men		
	No fracture	Fracture	*p* Value	No fracture	Fracture	*p* value
No. of people	1,956	67		1,018	18	
Age (years) in 2015	48 (14)	55 (13)	0.001	40 (14)	45 (14)	0.449
Age (years) at start HT	31 (24–41)	33 (26–45)	0.190	25 (21–33)	25 (21–34)	0.985
BMI (kg/m^2^) (*n* = 1785)	24.6 (4.4)	25.8 (5.1)	0.265	25.8 (4.9)	24.4 (3.5)	0.257
Smoking, % yes (*n* = 1714)	46	63	0.087	48	56	0.519
*T*‐score lumbar spine	−1.02 (1.28)	−1.34 (1.40)	0.021	−0.17 (1.19)	−0.43 (1.04)	0.973
*T*‐score total hip	−0.73 (0.86)	−0.94 (0.97)	0.175	+0.05 (0.98)	−0.35 (0.81)	0.204
*T*‐score femoral neck	−1.06 (0.92)	−1.36 (1.04)	0.073	−0.30 (1.05)	−0.79 (0.60)	0.138
Laboratory						
Estradiol (pmol/L)	220 (135–337)	172 (116–299)	0.585	148 (103–206)	84 (65–133)	0.030
Testosterone (nmol/L)	1.3 (0.8–1.3)	1.3 (0.9–2.2)	0.446	25 (17–37)	22 (17–29)	0.265
LH (IU/L)	2.5 (0.3–9.3)	2.8 (0.7–9.6)	0.808	3.6 (0.9–11.4)	10.1 (2.2–15.2)	0.231

HT = hormonal treatment; BMI = body mass index; LH = luteinizing hormone. Characteristics are shown as mean with standard deviation, median with interquartile range, or percentage.

Laboratory measurements were available for 66% of the trans women and 72% of the trans men.

### Trans men

Eighteen trans men experienced a fracture (1.7%) in the 3‐year time period, whereas 3.0% of the age‐matched reference men (*n* = 155) and 2.2% of the age‐matched reference women (*n* = 114) had a fracture (Fig. 1). The fracture risk was similar to age‐matched reference women (OR = 0.79, 95% CI 0.48–1.30) but lower compared with age‐matched reference men (OR = 0.57, 95% CI 0.35–0.94) (Fig. 2). Table 2 displays the differences in characteristics between trans men with and without fractures. In the multivariable analyses, no associations were found between fracture risk and age in 2015 (per year: OR = 1.02, 95% CI 0.97–1.07), age at start of HT (per year: OR = 1.00, 95% CI 0.93–1.07), mean BMI (per point: OR = 0.92, 95% CI 0.80–1.06), smoking (yes versus no: OR = 1.44, 95% CI 0.48–4.30), and *T*‐score of the LS (per 1.0 point: OR = 0.99, 95% CI 0.62–1.59). Because of the smaller number of individuals with laboratory measurements, these were not included in the multivariable analyses. However, in the univariable analyses, it was found that estradiol concentrations were associated with fracture risk (per 10 pmol/L: OR = 0.89, 95% CI 0.80–0.99), but testosterone (per 1 nmol/L: OR = 0.98, 95% CI 0.93–1.02) and LH (per 1 IU/L: OR = 1.03, 95% CI 0.98–1.08) concentrations were not.

Because of the low number of fractures in trans men, no stratification for age or type of fracture could be performed.

## Discussion

In this study, we found that fracture risk was higher in older trans women using long‐term HT compared with age‐matched reference men but similar to age‐matched reference women. In young trans women, fracture risk tended to be increased compared with age‐matched reference women but not compared with age‐matched reference men. In addition, the type of fractures differed in trans women compared with age‐matched reference men, with relatively more hip, spine, forearm, and humerus fractures. Fracture risk was not increased in young trans men using long‐term HT.

In trans women aged <50 years, fracture risk tended to be increased compared with age‐matched reference women but not compared with age‐matched reference men. Earlier studies found that trans women, also at younger ages, had a high prevalence of osteoporosis or low BMD even before hormonal treatment.^16^, ^32^ Although earlier short‐term and long‐term studies did not show a detrimental effect of HT on BMD,^18^, ^31^ higher fracture risk in young trans women compared with age‐matched reference women may be explained by lower initial BMD even before start of HT. In the general population, men have a higher fracture incidence than women at younger ages and these fractures usually occur as a result of an accident.^37^, ^38^ It can be speculated that, although BMD in trans women is lower than in control men, fracture risk is not increased because trans women often tend to have a less active lifestyle than control men and are therefore less likely to suffer an accident that leads to a fracture.

In trans women aged ≥50 years, fracture risk was increased compared with age‐matched reference men but similar to age‐matched reference women. At older ages, control women have a higher fracture risk than control men, mainly because of a decreased BMD in control women because of the loss of estrogen after menopause.^37^ The similar risk in trans women compared with age‐matched reference women at older ages can be thought to be the result of decreased estrogen concentrations, possibly because of decreasing or discontinuation of estradiol supplementation in older trans women. However, this is not part of our clinical protocol and we did not find a difference in mean estradiol concentrations in trans women aged <50 years and ≥ 50 years. Control women usually have normal BMD before menopause, but it decreases during menopause because the loss of estrogen. Trans women, however, have low BMD but, in our center, do not stop or lower estrogen therapy at the age of 50 years and therefore do not experience a decrease in BMD because of the loss of estrogen. This might explain why the fracture risk becomes similar in trans women compared with age‐matched reference women after the age of 50 years but higher than age‐matched reference men.

It was also found that the type of fractures differed in trans women compared with age‐matched reference men, with relatively more hip, spine, forearm, and humerus fractures, whereas it was similar to age‐matched reference women. This might be explained by the fact that hip, spine, forearm, and humerus fractures usually occur because of low BMD, whereas other fractures mostly occur because of accidents. Because trans women have lower BMD than control men, the risk of getting a hip, spine, forearm, or humerus fracture is higher, but because of a supposed less active lifestyle, the risk for other fractures is lower.

In trans women, lower *T*‐score of the LS was associated with an increased fracture risk. Of the analyzed DXA scans in trans women, 58% were performed at baseline and 42% were performed during HT. In trans women of whom baseline DXA scans were analyzed, the same differences in *T*‐scores of the lumbar spine were found between those with and without fractures compared with the trans women of whom DXA scans during HT were analyzed (data not shown). This is in line with the finding of an earlier study, describing that BMD does not change during long‐term HT.^31^ This finding indicates that the increased fracture risk in trans women is likely attributable to the low pretreatment BMD as described before,^31^, ^32^ instead of the effects of hormonal treatment.

Trans men had a similar fracture risk compared with age‐matched reference women but a lower risk than age‐matched reference men. Earlier studies found no increased fracture risk,^4^, ^20^, ^33^ a normal BMD at baseline,^20^ and no detrimental effects of HT on BMD.^20^, ^25^, ^31^ Therefore, it was not expected that fracture risk would be increased in trans men. The finding that trans men had a lower fracture risk than age‐matched reference men but similar to age‐matched reference women might be explained that trans men are more careful or participate less in (sporting) activities than the age‐matched reference men, leading to fewer fractures.

This study is the first to investigate the fracture risk in a large population of adult trans women and trans men using long‐term HT. This population was linked to a random sample of 10 age‐matched reference men and women per person, which makes the control population more accurate. The occurrence of fractures was retrieved from the database from Statistics Netherlands, which stores all diagnoses of the visits to the emergency rooms nationwide. As in the Netherlands, everybody with a (suspicion of a) fracture visits the emergency room, so this is a reliable source for fracture data. However, there are also some limitations. First, the time frame for fracture occurrence was only 3 years. Although it is long enough for a point prevalence of fractures, most ideally transgender people would be followed from the moment they start with HT. Second, we did not have any clinical data of the age‐matched reference population besides fracture data. Therefore, no other factors with effect on fracture risk, for example BMD, smoking habits, BMI, and physical activity, could be analyzed in the control groups. However, in a large population‐based study in the Netherlands, it was found that 48% of the cis women and 56% of the cis men are (former) smokers. The mean BMI is 25.6 kg/m^2^ in cis women and 25.8 kg/m^2^ in cis men.^39^ These numbers are comparable to the percentage of (former) smokers and mean BMI in our transgender population. In addition, in studies from our neighbor country Belgium, no differences were found in physical activity between the transgender population and control groups.^20^, ^32^ Third, this study used data that were collected during clinical care. Therefore, not all data were available from the entire population, such as BMD and laboratory measurements. However, because they were known for the majority of the included population, these data were still analyzed but should be interpreted cautiously. Fourth, because this study was performed in collaboration with Statistics Netherlands, it was only allowed to show data if more than 10 individuals per group were present to protect the anonymity of the data. Therefore, only the overall fracture risk could be shown in trans men. Fifth, only people with a suspicion of a fracture visit the emergency room. Because some fractures are asymptomatic, some fractures might have been missed. However, as this would occur in both the transgender as the control population, it probably would not influence the results of the analyses. Lastly, although the transgender population used HT for a long term, the population was still young of age. Because most fractures occur at higher age, only the fracture risk at younger ages was assessed in this study. The effects of HT on fracture risk at old age and the etiology of the fractures therefore remain topics for further research.

In conclusion, fracture risk was higher in older trans women using long‐term HT compared with age‐matched reference men but similar to age‐matched reference women. In young trans women, fracture risk tended to be increased compared with age‐matched reference women but not compared with age‐matched reference men. Fracture risk was not increased in young trans men using long‐term HT. However, because type of fractures differed in trans women and the transgender population was still young, bone health remains an important health topic, particularly in trans women, and longer follow‐up studies are needed.

## Disclosures

The authors have no relevant disclosures to declare.
